# L-Carnitine Prevents Behavioural Alterations in Ketamine-Induced Schizophrenia in Mice: Possible Involvement of Oxidative Stress and Inflammation Pathways

**DOI:** 10.1155/2023/9093231

**Published:** 2023-06-16

**Authors:** Mehrasa Ebrahimi, Nematollah Ahangar, Ehsan Zamani, Fatemeh Shaki

**Affiliations:** ^1^Pharmaceutical Sciences Research Center, Faculty of Pharmacy, Mazandaran University of Medical Sciences, Sari, Iran; ^2^Students Research Committee, Faculty of Pharmacy, Mazandaran University of Medical Sciences, Sari, Iran; ^3^Department of Pharmacology, School of Medicine, Guilan University of Medical Sciences, Rasht, Iran; ^4^Department of Pharmacology and Toxicology, School of Pharmacy, Guilan University of Medical Sciences, Rasht, Iran; ^5^Department of Toxicology and Pharmacology, Faculty of Pharmacy, Mazandaran University of Medical Sciences, Sari, Iran

## Abstract

Schizophrenia is a chronic mental complaint known as cognitive impairment. There has been evidence that inflammation and oxidative stress play a main role in schizophrenia pathophysiology. This study aimed to investigate the effects of l-carnitine, as a potent antioxidant, on the treatment of behavioural and biochemical disturbances in mice with ketamine-induced schizophrenia. In this study, schizophrenia was induced in mice by ketamine (25 mg/kg/day, *i.p*). Before induction of schizophrenia, mice were treated with l-carnitine (100, 200, and 400 mg/kg/day, *i.p*). Then, behavioural impairments were evaluated by open field (OF) assessment and social interaction test (SIT). After brain tissue isolation, reactive oxygen species (ROS), glutathione concentration (GSH), lipid peroxidation (LPO), protein carbonyl oxidation, superoxide dismutase activity (SOD), and glutathione peroxidase activity (GPx) were assessed as oxidative stress markers. Furthermore, inflammatory biomarkers such as tumour necrosis factor alpha (TNF-*α*) and nitric oxide (NO) were evaluated in brain tissue. Our results showed ketamine increased inflammation and oxidative damage in brain tissue that was similar to behaviour disorders in mice. Interestingly, l-carnitine significantly decreased oxidative stress and inflammatory markers compared with ketamine-treated mice. In addition, l-carnitine prevented and reversed ketamine-induced alterations in the activities of SOD and GPx enzymes in mice's brains. Also, improved performance in OFT (locomotor activity test) and SIT was observed in l-carnitine-treated mice. These data provided evidence that, due to the antioxidant and anti-inflammatory effects of l-carnitine, it has a neuroprotective effect on mice model of schizophrenia.

## 1. Introduction

Schizophrenia (SZ) is a severe, chronic, and debilitating psychiatric illness that affects about 1% of the world's population [1]. Positive (e.g., hallucinations and paranoia), negative (e.g., blunted affects and social withdrawal), and cognitive (e.g., executive and memory dysfunction) symptoms are hallmarks of the disease [2]. Many factors may contribute to the progression of this disorder, which can be pointed out to genetic, neurobiological, psychological, social, and environmental factors [3]. Several molecular mechanisms were suggested for this mental disorder, including alterations in neurotransmitter (such as dopaminergic and glutamatergic) system [4], alterations in nitric oxide (NO) signaling [5], upregulation of inflammation status, and oxidative stress [6]. However, the exact pathophysiology of SZ is yet to be determined.

The toxic role of free radicals in the etiology of SZ was proposed in the 1950s [7] and many research studies on the role of oxidative stress in SZ have been carried out. Oxidative stress occurs due to an increase in the level of reactive oxygen (ROS) and nitrogen (RNS) species or a decrease in the activity of the endogenous antioxidant system [8]. The brain is particularly susceptible to oxidative stress due to its high oxygen consumption, low antioxidant capacity (such as catalase, superoxide dismutase, etc.), high metabolic requirements, and high content of polyunsaturated fatty acids [9]. The relationship between SZ and oxidative stress was proven when the level and activity of antioxidants (such as glutathione, glutathione peroxidase, superoxide dismutase, and catalase) decreased in SZ models [10]. However, some studies have reported no change in these issues [11].

Inflammation is another pathogenic process that is suggested as the etiology of SZ [12]. Proinflammatory cytokines such as interleukins (ILs), interferons (IFNs), and tumour necrosis factors (TNFs) contribute to the normal function of the central nervous system (CNS) [13]. It was shown that abnormal levels of these cytokines can be considered one of the major causes of inflammatory abnormalities in SZ [14]. Since the immune hypothesis of SZ has been expressed in 1980s [15], a growing number of research investigations have developed around the immune system and the role of neuro-inflammation in patients with SZ.

Glutamatergic hypothesis, NMDA receptor antagonists, such as ketamine or phencyclidine are widely used for reproducing the behavioural (positive, negative, and cognitive) and biochemical symptoms that are associated with SZ in an animal model [16, 17]. Several studies showed the role of antioxidants for improving the outcome of SZ, such as folic acid [1], omega-3 [10], and *Spinacia oleracea* seed extract [18].

L-Carnitine (l-car) is required for *β*-oxidation of long-chain fatty acids in mitochondria [19]. It also showed antioxidant properties, such as boosting the antioxidant system [20], preventing lipid peroxidation [21], protecting DNA and cell membranes from damage caused by ROS [22, 23], helping to normalize age-related changes [24, 25], and also increasing the immune system's ability [26]. Furthermore, some recent studies have reported the anti-inflammatory [27] and neuroprotective [28, 29] effects of l-car and its positive effects on some diseases [30, 31].

Different models have been proposed for evaluating schizophrenic diseases until now [32]. Since the treatment of SZ is still a challenge, it would be worthwhile to find a new therapeutic strategy for it on the evidence that l-car can act as an antioxidant and anti-inflammatory agent in clinical and animal studies (fibrosis [33], hemodialysis [34], heart disease [35], Huntington [36], etc.).

This research was designed to evaluate the effect of l-car on behavioural changes as well as its protective effect in preventing inflammation and oxidative damage in the brain of a mouse model of SZ.

## 2. Materials and Methods

### 2.1. Chemicals

Ketamine, l-carnitine, xylazine, mannitol, DCFH-DA (2′,7′-dichlorofluorescein diacetate), DNPH (2,4-dinitrophenylhydrazine), Tris-HCl, tris ammonium, bovine serum albumin (BSA), MgCl_2_, disodium hydrogen phosphate, tricarboxylic acid, sucrose, EDTA, sodium acetate, and Triton X-100 were from Sigma; phosphate buffered saline (PBS) was from Gibco. Phosphoric acid, methanol, Coomassie Brilliant Blue, acetic acid glacial, potassium chloride, n-butanol, sodium chloride, DTNB, sodium hydroxide, and DMSO were from Merck.

### 2.2. Animals

In this study, adult male mice (25–30 g) were used. All procedures were conducted based on the ethical standards and protocols approved by the institutional animal care of Mazandaran University of Medical Sciences, Sari, Iran, and are consistent with the NIH Guide (Ethical code: IR.MAZUMS.REC.1398.105). Animals were kept under standard conditions (12-h light/dark cycle, temperature of 22 ± 3°C, and 85% relative humidity) with adequate access to food and water.

### 2.3. Experimental Design

**Figure 1:**
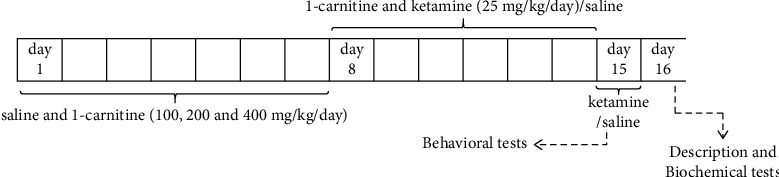
Diagram of the complete experimental schedule.

Thirty mice were randomly separated into 5 groups (*N* = 6) as follows: control group received 0.9% NaCl; ketamine group received saline for the first seven days, and then ketamine (25 mg/kg/day) was administered in the next seven days to create some of the psychological symptoms found in SZ based on previous reports [1]; l-car + ket group received different doses of l-car (100, 200, and 400 mg/kg/day) for 14 days plus ketamine (25 mg/kg/day) [33, 37].  Figure 1 shows the experimental schedule. All compounds were administered via the intraperitoneal route (*i.p*.). On the 15th day of treatment, ketamine or saline was injected 30 minutes before behavioural tests to create an animal model of SZ. One day after the last injection, animals were killed by ketamine/xylazine. Brain tissues were dissected carefully for the biochemistry analysis.

### 2.4. Behavioural Evaluation

#### 2.4.1. Locomotor Activity Test

The exploratory behaviour was measured by an open-field apparatus. The computer defined grid lines that divided the open field into 25 squares of equal area. The mice were placed in an open field (50 × 25 × 50 cm) with black walls and floor. Then, all observers left the place in order to let animals freely explore the arena for 5 minutes. The animal's activities were tracked and recorded by a camera. Thus, the direction of the mouse movement was recorded at each training time, and the total distance travelled was measured. After every trial, the device was cleaned up with 70% ethanol and dried [38].

#### 2.4.2. Social Interaction Test

Negative symptoms were assessed by a social interaction test. Immediately after the locomotor activity test, each animal was isolated for 6 hours without any access to water and food before experimentation. Then, 2 animals were randomly selected from a group and placed in the open field arena (50 × 25 × 50 cm) for 15 minutes. For each individual animal, three parameters were evaluated during this period: the latency to the first contact between animals, number of contacts between animals, and the interaction time the animals stay together [1].

### 2.5. Oxidative Stress Evaluation

#### 2.5.1. Preparation of Samples

24 h after the last injection, mice were euthanized by injection of ketamine (80 mg/kg) and xylazine (5 mg/kg). The brain tissues were scraped off and fragmented by scissors. After washing with cold mannitol buffer, tissues were homogenized in the buffer (0.255 M mannitol, 74 mM sucrose, 0.2 mM EDTA) and then centrifuged for 10 minutes at 1400 rpm. At the end, the supernatant was used to measure biochemical parameters.

Furthermore, the protein content of samples was determined via the Bradford method [39].

#### 2.5.2. Evaluation of ROS Generation

DCFH-DA (2′,7′-dchlorofluorescein diacetate) was used as an indicator for the evaluation of ROS generation. Briefly, 200 *μ*l DCFH (final concentration 10 *μ*M) was added to 2 ml of brain tissue homogenates (1 mg protein/ml) and incubated for 15 min at 37. Then, its fluorescence intensity was examined at 480 nm (excitation) and at 520 nm (emission) by a Shimadzu RF5000 U fluorescence spectrophotometer [40].

#### 2.5.3. Lipid Peroxidation Assay

Lipid peroxidation was analyzed using the thiobarbituric acid-malondialdehyde (TBA-MDA) assay [40]. Briefly, brain homogenate was mixed with 0.25 ml of phosphoric acid (0.05 M) and 0.3 ml of 0.2% TBA and then heated in a boiling water bath for 30 min. After cooling by ice bath, 0.4 ml n-butanol was added, and supernatants from centrifuging (3500 rpm/10 min) were collected. Eventually, the pink chromogen was evaluated at 532 nm via an ELISA plate reader (Tecan, Rainbow Thermo, Austria). Tetra-methoxy-propane concentrations were used to prepare the standard curve.

#### 2.5.4. Determination of Glutathione Content

Glutathione (GSH) content, an intrinsic cellular antioxidant, was evaluated based on Ellman's reagent (DTNB) reaction with free thiol groups [41]. Briefly, 100 *μ*l of sample was added to 0.1 ml of phosphate buffer and 0.04% DTNB (5, 5′-dithio-bis-(2-nitrobenzoic acid)) in a total volume of 0.3 ml (PH 7.4). Finally, yellow color was assessed by a spectrophotometer (UV-1601 PC, Shimadzu, Japan) at 412 nm.

#### 2.5.5. Evaluation of Protein Carbonyl Concentration

Protein carbonyl was evaluated by using the Dalle-Donne method [42], in which carbonylated protein forms a chromophoric adduct with 2,4-dinitrophenylhydrazine (DNPH). The absorption of the yellow product was read at 365 nm on a spectrophotometer. Results were displayed as mM of carbonyl content per mg of protein.

### 2.6. Evaluation of Antioxidant Enzyme Activity

#### 2.6.1. Evaluation of Superoxide Dismutase Activity

The determination of superoxide dismutase (SOD) was performed by the SOD activity assay kit (ZellBio, Germany, Cat No. ZB-SOD-96A) based on the kit's protocol. In this kit, the superoxide anion was used for conversion to hydrogen peroxide and oxygen under enzymatic reaction conditions. Finally, the chromogenic product was made, which was measured colorimetrically at 420 nm [43]. SOD activity was calculated in the corresponding formula and expressed as U/ml

(1) $$\begin{array}{c} Vp={OD}_{sample\;2\min}-{OD}_{blank\;2\min}\text{,}\\ Vc={OD}_{Sample\;0\min}-{OD}_{blank\;0\min}\text{,}\\ SOD\;activity\left(\frac{U}{mL}\right)=\frac{\left(Vp-Vc\right)}{\left(Vp\right)}\times 100\text{.} \end{array}$$

#### 2.6.2. Evaluation of Glutathione Peroxidase Activity

Glutathione peroxidase activity (GPx) was evaluated by using the GPx activity assay kit (ZellBio, Germany, Cat No. ZB-GPX-96A) according to the company's instructions on the basis of the colorimetric assay (412 nm). GPx uses GSH as the ultimate electron donor to regenerate the reduced form of selenocysteine. By addition of excess GSH, GPx converts it to GSSG, and remaining GSH can be reduced and generate yellow color by reducing DTNB (at 412 nm). The GPx activity is indirectly related to color formation [44]. The activity of GPx was estimated using the given formula, and U/ml was reported

(2) $$\begin{array}{c} GPx\;activity\left(\frac{U}{mL}\right)=\frac{\left({OD}_{control}-{OD}_{sample}\right)}{\left({OD}_{standard}-{OD}_{blank}\right)}\times 6000\text{.} \end{array}$$

### 2.7. Inflammatory Marker Evaluation

#### 2.7.1. Nitric Oxide (NO) Determination

The commercial kit based on the Griess reagent (Cib-Biotech Company, Iran, Cat No. 3201−200) was used for the estimation of nitric oxide content in brain tissue. In this method, diazonium ion was formed by the reaction of nitrite and sulfanilic acid. Azo pink derivatives were formed by connecting the ion to *N*-(1-naphthyl) ethylenediamine, which was measured through a spectrophotometer at 548 nm. The concentration of nitrite was determined by a standard curve of 0.1 M sodium nitrite in distilled water and was expressed as µmol/mg of protein [45].

#### 2.7.2. Assay of TNF-Alpha Concentration

Levels of TNF-*α* in brain tissue were measured by Diaclone kits (Diaclone, France, Cat No. 865000096) based on the company's instructions at 450 nm. The TNF-*α* concentration of the unknown samples was calculated from the standard curve of the standard cytokine [46].

### 2.8. Statistical Analysis

All results are expressed as mean ± SD. The distribution of our data follows a normal pattern. All statistical analyses were performed using GraphPad Prism® software (version 6). Data were analyzed by one-way ANOVA with *p*  <  0.05 as the level of significance. When ANOVA showed a significant difference, Tukey's post hoc test was applied.

## 3. Results

### 3.1. Results of Behavioural Evaluation

#### 3.1.1. The Effect of L-Carnitine on Locomotor Activity

**Figure 2:**
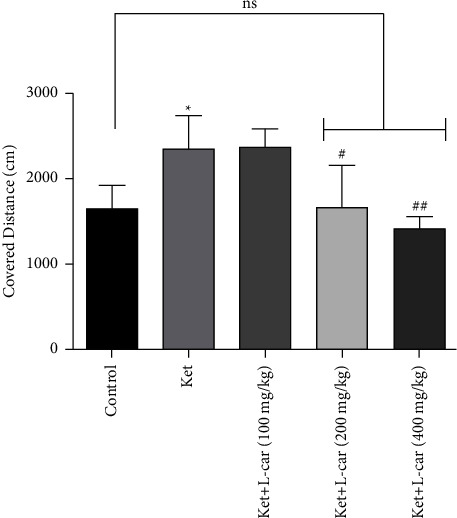
Effect of l-carnitine (l-car) administration (100, 200, and 400 mg/kg) for a period of 14 days on locomotor activity induced by ketamine (ket) in mice (25 mg/kg/day for 7 days); data are expressed as mean ± SD (*n* = 6). ^*∗*^*P* <  0.05 compared to control; ^#^*P* < 0.05 compared ket; ^##^*P* < 0.01 compared to ket; ns: no significant difference.

Based on Figure 2, the total distance travelled by the animal significantly increased in the ketamine group compared to the control group (*P* <  0.05), which indicates anxiety-like behaviour. In the pretreatment group with 200 and 400 mg/kg of l-car, total mileage significantly decreased compared to the ketamine group (from low to high concentrations: *P* <  0.05 and *P* <  0.01), which indicates the anxiolytic effect of l-car. So, l-car pretreatment at these doses was notable for preventing the hyperlocomotion induced by ketamine in the open-field test.

#### 3.1.2. The Effect of L-Carnitine on Social Interaction Test

**Figure 3:**
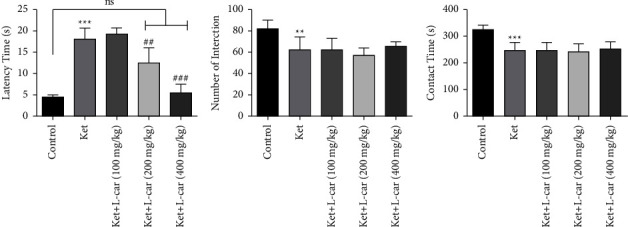
Effect of l-carnitine (l-car) administration (100, 200, and 400 mg/kg) for a period of 14 days on social interaction test in schizophrenic mice induced by ketamine; (a) latency to the first contact; (b) number of interactions; (c) total contact time. Data are expressed as mean ± SD (*n* = 6). (a) ^*∗∗∗*^*P* <  0.001 compared to control; ^##^*P* <  0.01 compared to ket; ^###^*P* < 0.001 compared to ket; ns: no significant difference. (b) ^*∗∗*^*P* < 0.01 compared to control. (c) ^*∗∗∗*^*P* < 0.001 compared to control.

According to Figure 3(a), ketamine (25 mg/kg) significantly increased the time of the first connection between animals compared with the control group (*P* <  0.001), so it caused social defects. However, l-car at doses of 200 and 400 mg/kg during the treatment period completely prevented an increase in the latency time induced by ketamine (*P* <  0.01 and *P* <  0.001, respectively). As a result, pretreatment at these doses of l-car has a protective effect against the increased delay in the first connection between the animals caused by ketamine.

As shown in Figure 3(b), the number of social contacts was significantly decreased in the ketamine group when compared to the control (*P* <  0.01). In contrast, two-week administration of l-car (400 mg/kg) partially increased the number of contacts which is not significant.

Figure 3(c) shows the duration of communication in the ketamine group was significantly lower than the control group (*P* <  0.001). No significant differences were observed in total interaction time between the ketamine group and the l-car group, indicating no protective effect of these doses of l-car on reducing the total time of social contacts induced by ketamine.

### 3.2. Results of Oxidative Stress Evaluation

#### 3.2.1. Effects of L-Carnitine on ROS Generation

**Figure 4:**
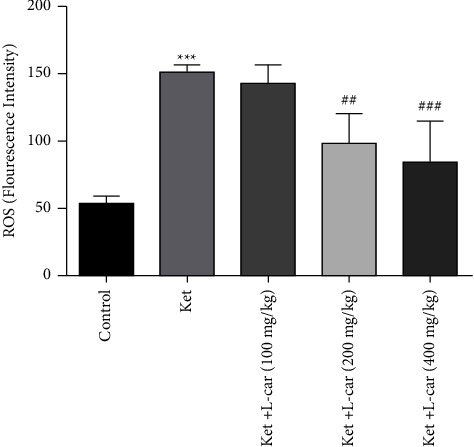
Effects of l-carnitine (l-car) administration (100, 200, and 400 mg/kg) on ROS formation in the brain of schizophrenic mice induced by ketamine (ket); data are expressed as mean ± SD (*n* = 6). ^*∗∗∗*^(*P*) <  0.001 compared to control. ^##^*P* < 0.01 compared to ket; ^###^*P* < 0.001 compared to ket.

As presented in Figure 4, the highest levels of ROS production were observed in the ketamine group and the lowest in the control group. The results showed that the amount of ROS production in the ketamine group was significantly elevated compared with the control group (*P* <  0.001). In contrast, this level was significantly lower in the groups receiving l-car at doses of 200 and 400 mg/kg compared to the ketamine group (*P* <  0.01 and *P* <  0.001, respectively). In fact, administration of these two doses of l-car showed a neuroprotective effect against the production of free radicals induced by ketamine, whereas l-car pretreatment at low doses had no significant effects on ketamine-induced ROS formation.

#### 3.2.2. Effects of L-Carnitine on Lipid Peroxidation

**Figure 5:**
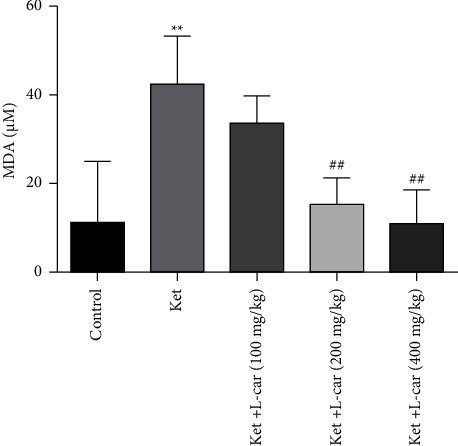
Effects of l-carnitine (*l*-car) administration (100, 200, and 400 mg/kg) on lipid peroxidation in the brain of schizophrenic mice induced by ketamine (ket); data are expressed as mean ± SD (*n* = 6). ^*∗∗∗*^(*P*) <  0.01 compared to control. ^##^*P* < 0.01 compared to ket.

According to Figure 5, the highest concentration of malondialdehyde, an end product of lipid peroxidation, was observed in the ketamine group. The level of MDA in the ketamine group was significantly higher than that in the control group (*P* <  0.01). The concentration of MDA in the l-car groups (200 and 400 mg/kg) showed a significant reduction (*p*  <  0.01) as compared to the ketamine group. The reduction of MDA in the l-car group at a dose of 100 mg/kg was not significant. Increasing the transmission of fatty acids to mitochondria and reducing the fatty acids exposed to peroxidation seem to be the main reasons for the reduction of MDA in the brain.

#### 3.2.3. Effects of L-Carnitine on Glutathione Content

**Figure 6:**
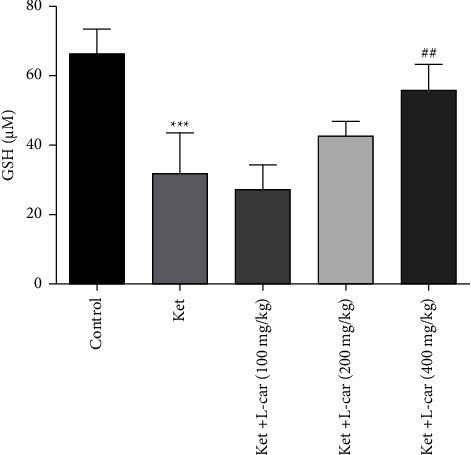
Effects of l-carnitine (l-car) administration (100, 200, and 400 mg/kg) on GSH contents in the brain of schizophrenic mice induced by ketamine (ket); data are expressed as mean ± SD (*n* = 6). ^*∗∗∗*^(*P*) <  0.001 compared to control. ^##^*P* < 0.01 compared to ket.

As demonstrated in Figure 6, the GSH content was significantly decreased in ketamine-administered mice compared with the control group (*P* <  0.001). Pretreatment with 200 and 400 mg/kg of l-car increased GSH content compared to the ketamine group, but this increase was only significant at the dose of 400 mg/kg (*P* <  0.01). In contrast, l-car (100 mg/kg) was not able to reverse the reduction in GSH content.

#### 3.2.4. Effects of L-Carnitine on Protein Carbonyl Concentration

**Figure 7:**
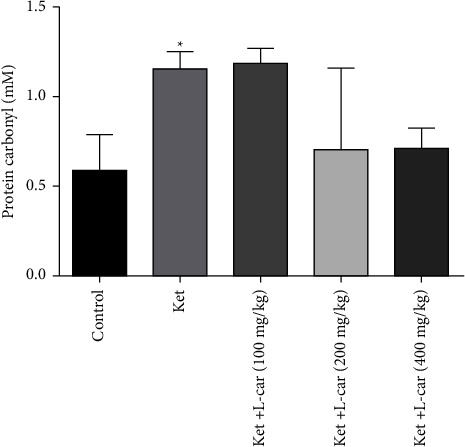
Effects of l-carnitine (l-car) administration (100, 200, and 400 mg/kg) on protein carbonyl levels in the brain of schizophrenic mice induced by ketamine (ket); data are expressed as mean ± SD (*n* = 6). ^*∗*^*P* <  0.05 compared to control.

Figure 7 shows a significant increase in protein damage induced by ketamine administration in the brain. The concentration of carbonyl protein in the ketamine group was significantly elevated when compared to the control group (*P* <  0.05). Pretreatment with 200 and 400 mg/kg of l-car resulted in a reduction of ketamine-induced protein carbonylation, but it was not statistically significant. No significant change was observed in the carbonylated protein concentration in l-car (100 mg/kg) administration compared to the ketamine group.

### 3.3. Results of Antioxidant Enzyme Activity

#### 3.3.1. Effects of L-Carnitine on SOD Activity

**Figure 8:**
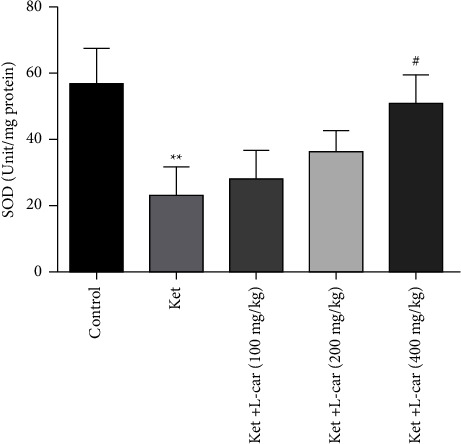
Effects of l-carnitine (l-car) administration (100, 200, and 400 mg/kg) on SOD activity in the brain of schizophrenic mice induced by ketamine (ket); data are expressed as mean ± SD (*n* = 6). ^*∗∗*^(*P*) <  0.01 compared to control; ^#^*P* <  0.05 compared to ket.

As depicted in Figure 8, the level of enzyme activity of SOD decreased significantly in the ketamine group when compared with the control group (*P* <  0.01). However, the level of SOD activity in the l-car (400 mg/kg) group showed a significant increase as compared to the ketamine group (*P* <  0.05). Increasing SOD activity in other l-car groups is not meaningful.

#### 3.3.2. Effects of L-Carnitine on Glutathione Peroxidase Activity

**Figure 9:**
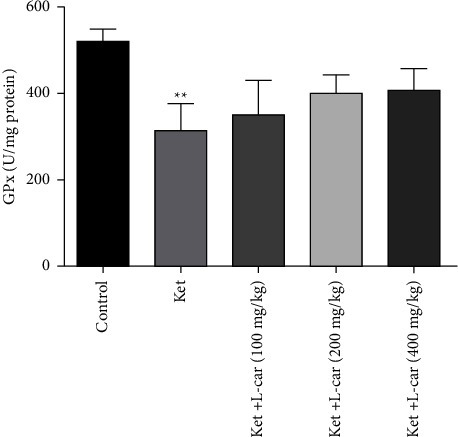
Effects of l-carnitine (l-car) administration (100, 200, and 400 mg/kg) on GPx activity in the brain of schizophrenic mice induced by ketamine (ket); data are expressed as mean ± SD (*n* = 6). ^*∗∗*^*P* <  0.01 compared to control.

As shown in Figure 9, the level of GPx activity in the ketamine group was significantly reduced compared to the control group (*P* <  0.01). In contrast, the activities of this enzyme in all groups of l-car were increased as compared with the ketamine group, which is not significant.

### 3.4. Results of Inflammatory Markers Evaluation

#### 3.4.1. Effects of L-Carnitine on NO Levels

**Figure 10:**
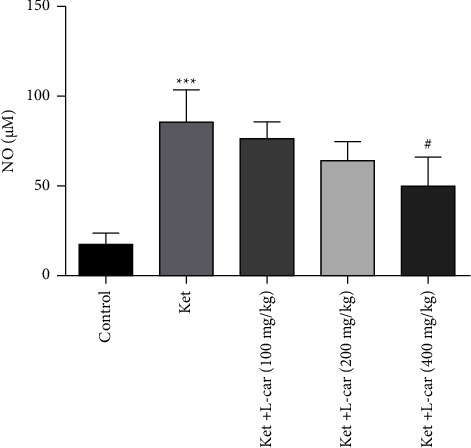
Effects of l-carnitine (l-car) administration (100, 200, and 400 mg/kg) on NO levels in the brain of schizophrenic mice induced by ketamine (ket); data are expressed as mean ± SD (*n* = 6). ^*∗∗∗*^*P* <  0.001 compared to control; ^#^*P* <  0.05 compared to ket.

As presented in Figure 10, the levels of NO in the ketamine group were significantly increased as compared to the control group (*P* <  0.001). On the other hand, the different doses of l-car prevented ketamine-induced increases in the level of NO; this decrease was only significant at the dose of 400 mg/kg (*P* <  0.01). Therefore, 400 mg/kg of l-car is more effective for reducing the NO brain level.

#### 3.4.2. Effects of L-Carnitine on TNF-*α* Levels

**Figure 11:**
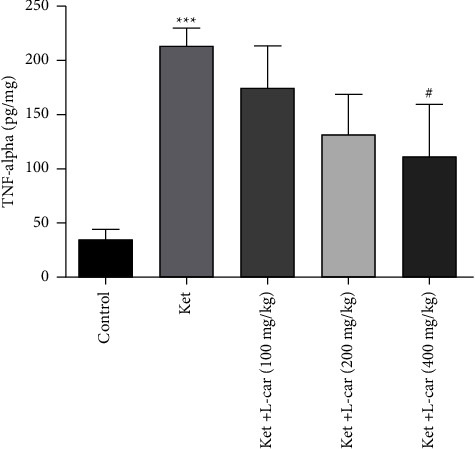
Effects of l-carnitine (l-car) administration (100, 200, and 400 mg/kg) on TNF-*α* levels in the brain of schizophrenic mice induced by ketamine (ket); data are expressed as mean ± SD (*n* = 6). ^*∗∗∗*^*P* <  0.001 compared to control. ^#^*P* <  0.05 compared to ket.

As demonstrated in Figure 11, the levels of TNF-*α*, the proinflammatory cytokine, were significantly increased in ketamine group compared with the control (*P*  <  0.001). All doses of l-car were found to reduce inflammation by decreasing the levels of TNF-*α* in the brain as compared to the ketamine group, but this decrease was only significant at the highest dose (*P*  <  0.05). Thus, l-car (400 mg/kg) was able to prevent ketamine-induced neuro-inflammation.

## 4. Discussion

In this study, we investigated the protective effect of three different doses of l-car against biochemical and behavioural disorders induced by intraperitoneal injection of ketamine in mice. The results showed that treatment with l-car for 14 days, especially at a dose of 400 mg/kg, significantly decreased the behavioural changes and the level of inflammatory and oxidative markers created by ketamine.

It is well known that chronic administrations of NMDA receptor antagonists (ketamine) can induce the core symptoms of SZ, in healthy humans and animals, and exacerbation of symptoms, in schizophrenic patients [16, 47, 48]. Animal models are a useful tool for studying a wide range of diseases. Therefore, ketamine is used in this study to model SZ in mice. Additionally, our experiments extended these data, which reinforced that ketamine can generate behavioural and neurochemical alterations relevant to the SZ for modelling the symptoms of this illness.

In the present study, behavioural data represented that hyperlocomotion, as a positive symptom, and social deficits, as a negative symptom, were induced by nonaesthetic doses of ketamine. These findings matched previous studies [1, 10, 18, 49, 50]. Also, it was revealed that l-car normalized ketamine-induced behavioural changes related to SZ. This result is similar to those of Vamos et al. [36], who showed that intraperitoneally administered l-car significantly ameliorated motor activity as compared with the control Huntington's disease transgenic mouse model group. In this regard, the anticonvulsant or antiepileptic effect of l-car has been identified by some animal studies [51, 52]. Supporting our data, evidence available has shown that acetyl-l-carnitine (ALCAR) improved the learning ability of elderly mice [53], recovered pathological and behavioural impairments in neurodegenerative disorders such as Alzheimer [54, 55], and could suppress anxiety [56] and depression [57] in preclinical studies. Moreover, Scafidi et al. [58] concluded that treatment with l-car during the first 24 h after traumatic brain injury improved behavioural outcomes and reduced brain lesion volume in immature rats within the first 7 days after injury.

Recent studies have reported that free radicals increase in schizophrenic patients, and there is a positive relationship between oxidative stress and the severity of symptoms relevant to the SZ in these patients [59]. Studies have proved that ketamine may lead to increased dopamine activity due to its reaction with catecholamine. The self-oxidation effect of dopamine also produces free radicals and ultimately induces oxidative stress [60]. As shown in the results, ketamine administration led to increased ROS formation, LPO, and protein carbonylation and decreased GSH, SOD, and GPx in brain tissue and was parallel to behavioural alterations in mice. Relevant surveys proved that ketamine single administration (5, 10, and 20 mg/kg) on mice increased the content of nitrite oxide and LPO and decreased the level of GSH in the brain [61]. Another study indicated that intraperitoneal injection of ketamine at a dose of 25 mg/kg for 8 consecutive days reduces the activity of the SOD and catalase enzymes in the hippocampus [62]. Also, the findings of De Oliveira et al. are consistent with the results of our study, which reported that ketamine increased LPO, caused oxidative damage to proteins and DNA, and decreased the activity of catalase and SOD enzymes [63]. Interestingly, examining 30 schizophrenic patients demonstrated the involvement of oxidative stress as well as inflammation in the etiology of the disease [64]. In addition, behavioural symptoms of SZ can be a result of pro-oxidant and inflammatory alterations [65, 66]. In fact, excessive oxidative damage can lead to chronic inflammation [14]. Research studies consider inflammation as a double-edged sword that means inflammation itself is beneficial in the body, but it can be harmful if chronically induced and not controlled properly [67].

Based on the neuroinflammatory hypothesis, immune dysfunction and inflammation in the CNS could have a role in the pathogenesis of neurodegenerative disorders such as schizophrenia [68]. Furthermore, neuropathological studies have revealed that prolonged microglial activation and dysfunction may lead to neuronal apoptosis and damage, which are seen in neurodegenerative disorders such as schizophrenia [69]. The microglial activation (M1/M2 phenotypes) may lead to a binary activation profile of peripheral monocytes [70]. The M1 phenotype rises in response to inflammation and goes along with the release of TNF‐*α*, IL‐6, IL‐1*β*, ROS, and glutamate, whereas the M2‐type microglia help to resolve the inflammatory response and express IL‐4, IL‐13, IL‐25, IL‐1ra, insulin‐like growth factor 1, and COX1 [71].

Also, hypoglutamatergic conditions and impaired *N*-methyl-d-aspartate (NMDA) signaling play an important role in the pathophysiology of schizophrenia. Studies showed that NMDA antagonists such as phencyclidine (PCP) and ketamine induce microglial activation. Therefore, NMDA antagonists suggest an appropriate animal model of schizophrenia [69].

Ketamine has modulatory effects on inflammatory pathways [72]. This NMDA-receptor antagonist modulates the inflammation by interacting with inflammatory cells, cytokine production, and inflammatory mediators. Some research studies showed anti-inflammatory effects of ketamine [73, 74], while in other studies, ketamine was introduced as an inflammatory inducer [75–77]. Interestingly, ketamine's effect on the level of inflammatory cytosine such as TNF-*α* varies under different conditions, depending on the dose and duration [72]. On the other hand, an imbalance between proinflammatory and anti-inflammatory cytokines has been described in psychiatric disorders such as schizophrenia. Furthermore, ketamine administration can cause increased levels of proinflammatory cytokines in the cerebellum, which leads to behavioural manifestations such as schizophrenia and autism [75].

Our results revealed an elevated level of NO and TNF-*α* in mice receiving ketamine, which confirmed the role of inflammation in the etiology of this disease. In confirmation of this statement, several studies demonstrated that TNF-*α* [78, 79] and IL-6 [78, 79] concentrations increased in schizophrenic patients. Also, the levels of TNF-*α* have increased with ketamine in previous animal studies [18, 80].

The hypothesis that the l-car may help to cure the schizophrenic symptoms is based on the observation of its antioxidant and anti-inflammation attributes [81, 82]. As far as we know, there is no evidence about the effects of l-car on SZ, but the neuroprotective effects of carnitines in different conditions of metabolic stress have been reported [83]. L-car not only affects the transmission of fatty acids into the mitochondria as an antioxidant, it can also increase the activity of enzymatic (CAT, GPx, and SOD) and the level of nonenzymatic (vitamins C, E, and GSH) antioxidants [84]. As shown in the results, l-car significantly inhibited ketamine-induced ROS formation in brain tissue. On the other hand, LPO starts with free radicals (ROS) attacking fatty acids or their lateral branches [85]. L-car markedly decreased the concentration of MDA (as the final product of LPO [8]) due to ketamine administration. Also, oxidative stress causes oxidation of proteins with lateral lysine, arginine, and threonine amino acids and the carbonyl group produces in the lateral branch, which results in the loss of protein function [86]. Moreover, l-car improved the levels of cellular enzymatic (SOD and GPx) and nonenzymatic (GSH) antioxidants after ketamine administration. A recent study by Mescka et al. concluded that l-car reduced ROS, LPO, and carbonyl levels and increased GSH content, SOD and GPx activity, in some animal brain structures in a maple syrup urine disease model [87]. These findings are in good agreement with the results of this study. Also, Rani et al. demonstrated that the status of glutathione, ascorbic acid, and vitamin E decreased in the brains of aged rats, and adding l-car to their diet improved their antioxidant status, which resulted in decreased LPO and carbonyl content [24]. Another correlated study reported by Ozmen et al. which suggested the l-car was able to increase the activities of SOD and GSH but decrease LPO and neuronal degeneration in the formaldehyde hippocampal damage model [88].

NO acts as a key mediator of inflammation [89] and can regulate the expression of proinflammatory cytokines (such as TNF-*α*) [90]. In contrast, l-car reduces inflammation and the infiltration of neutrophils [37]. In this study, l-car also reversed the increased levels of NO and TNF-*α* induced by ketamine. A similar conclusion was reached by Görür et al. who demonstrated that l-car can prevent NO elevation in renal ischemia-reperfusion injury in the rat [37]. Another parallel study performed by Suchitra et al. proved the significant benefit of l-car supplementation on inflammatory status in hemodialysis patients, as noted by a marked decrease in hsCRP levels in comparison with the control group [81]. Also, Abd-Allah et al. showed that administration of l-car returned testicular NO and serum IL-2 to normal levels in a lipopolysaccharide model of sepsis [82]. Another evidence indicated that l-car supplementation has beneficial effects on reducing TNF-*α* levels in knee osteoarthritis patients [91]. More correlational evidence pointed out that l-car administration in drinking water significantly decreased the bleomycin-induced elevations of serum TNF-alpha and LPO level in lung tissues [92]. The results of Mustafa et al. which showed that l-car improved cardiac functions by reducing serum interleukin-1 beta (IL-1*β*) and TNF-*α*, alleviating oxidative stress, decreasing cardiac MDA and NO, and restoring cardiac-reduced GSH levels to normal levels [93], are broadly in accordance with the present results.

## 5. Conclusion

As observed in our results, we can confirm the fundamental role of oxidative stress and inflammation in the pathogenesis of ketamine-induced SZ. Furthermore, pretreatment with l-car can be useful in the treatment of the experimental model of SZ. Probably, the ameliorative effect of l-car on schizophrenic symptoms is related to its antioxidant activity, which reduces free radicals or amplifies the antioxidant system. It also has a protective effect on the inflammatory marker of TNF-*α*, possibly as an anti-inflammatory agent, and can improve the immune system and inflammation of SZ patients. In conclusion, l-car can attenuate that ketamine induced various behavioural and biochemical changes similar to SZ in mice. Hence, it can be suggested that l-car can be used to treat similar pathological conditions caused by oxidative stress and inflammation in clinical and preclinical studies.

## Data Availability

Data used in this study are available from the corresponding author upon reasonable request.
